# Diagnosis, pathomechanisms and therapy of cerebral amyloid angiopathy-related inflammation (CAA-ri)

**DOI:** 10.1186/s42466-025-00382-3

**Published:** 2025-04-26

**Authors:** Rebecca M. Seifert, Randolf Klingebiel, Wolf-Rüdiger Schäbitz

**Affiliations:** 1 Universitätsklinik für Neurologie, Evangelisches Klinikum Bethel, Bielefeld, Germany; 2 Institut für diagnostische und interventionelle Neuroradiologie, Evangelisches Klinikum Bethel, Bielefeld, Germany

**Keywords:** Neuroinflammation, Amyloid, Cerebral amyloid angiopathy-related inflammation, Amyloid-related imaging abnormalities, Magnetic resonance imaging, Modified Boston criteria

## Abstract

**Background:**

Research of the past years has refined our perception of cerebral amyloid angiopathy-related inflammation (CAA-ri) as a subacute autoimmune encephalopathy, which is presumably caused by elevated CSF concentrations of anti-amyloid β (Aβ) autoantibodies. A broad understanding of the pathophysiological mechanisms and diagnostic criteria of CAA-ri may lay the foundation for improved immunosuppressive treatment of the disease.

**Main text:**

Spontaneous CAA-ri mainly occurs in elderly patients but might also be evoked iatrogenically by modern treatment with amyloid-modifying therapies in Alzheimer’s disease (AD). On a histopathological level, CAA-ri is characterized by microglial activation and the formation of vasogenic edemas. Clinically, the disease frequently presents with progressive cognitive decline, focal neurological deficits, headache and epileptic seizures. While brain biopsy has formerly represented the gold standard in the diagnosis of CAA-ri, its importance has been increasingly replaced by clinical as well as radiological diagnostic criteria and the relevance of anti-Aβ autoantibodies in the CSF of affected patients. Though relevant progress has been achieved in immunosuppressive treatment of CAA-ri, the protocols lack standardization as well as decision criteria for the choice of the respective immunosuppressive agent.

**Conclusions:**

CAA-ri gains increasing interest as a spontaneous human model of iatrogenic edematous amyloid-related imaging abnormalities (ARIA-E) in the context of amyloid-modifying therapies. In near future, screening of AD patients for the presence of CAA-ri using CSF anti-Aβ autoantibodies might play a decisive role in the risk stratification as well as dosage finding of amyloid-modifying therapies, as they show high specificity for CAA-ri. The clinical and radiological diagnostic criteria by Auriel et al. allow diagnosis of probable resp. possible CAA-ri with high accuracy. Though only tested in small, specialized patient cohorts to date, additional imaging modalities (^11^C-PK11195 PET) might play a future role in the clinical monitoring of CAA-ri. Therapy of CAA-ri frequently encompasses initial steroid treatment, whereby different schemes, dosages as well as substances are used. Choice of immunosuppressive agents with higher potency still requires objective decision criteria, which should be established in future studies involving larger CAA-ri patient cohorts.

## Background

Impairment of cerebral vessel integrity may cause deleterious brain alterations including macro- and microbleeds, rapid cognitive decline, transient neurological symptoms and epileptic seizures [1, 2, 6, 8, 13, 15, 17, 18, 20, 23, 24, 27, 30, 40, 45, 47, 54, 61, 63, 64]. In this context, cerebral amyloid angiopathy (CAA) is of paramount importance. CAA is characterized by pathological deposition of amyloid β (Aβ) peptides within the walls of small to medium-sized arteries, arterioles and capillaries of the cerebral cortex and overlying leptomeninges [15, 66]. In the absence of neuropathological confirmation, the diagnosis of CAA is based on characteristic MR imaging findings summarized in the modified Boston criteria [15, 66].

**Fig. 1:**
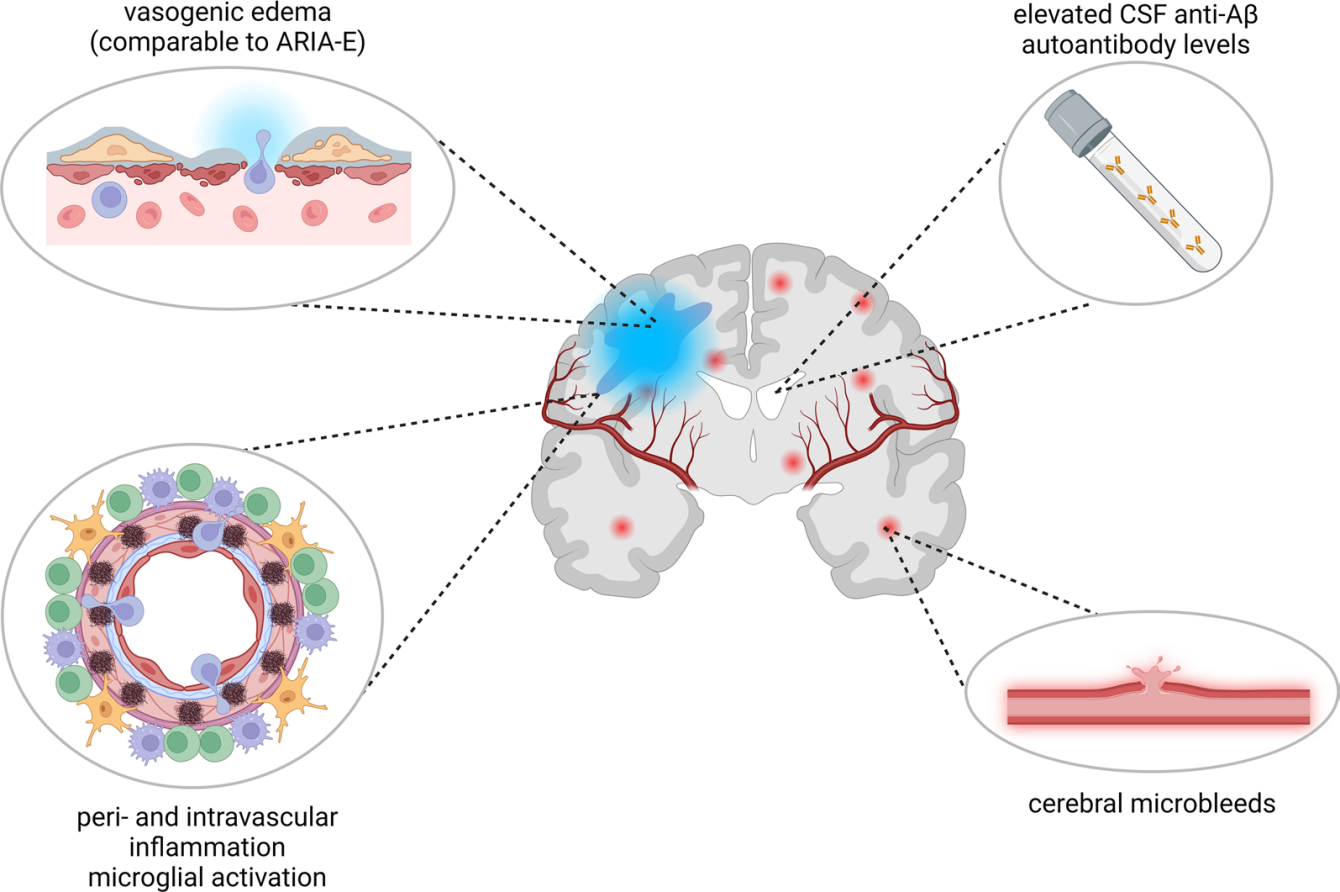
The pathomechanisms behind CAA-ri. In CAA-ri, anti-Aβ autoantibodies produced in the CSF of affected patients cause various autoinflammatory processes. Due to peri- and intravascular inflammation following microglial activation, vasogenic edemas comparable to iatrogenic ARIA-E are favored. CAA-ri also increases the risk for cerebral micro- as well as macrobleeds. Figure created with BioRender.com

Besides this vascular pattern of damage, various authors have occasionally described a concomitant inflammatory reaction adjacent to some amyloid-laden vessels seen in CAA [17, 18, 21, 26, 43, 48, 62]. Hence, this autoinflammatory disease nowadays known as cerebral amyloid angiopathy-related inflammation (CAA-ri) was initially considered as an inflammatory CAA subtype. However, further studies could show occurrence of CAA-ri also independent of underlying CAA [1, 12, 23, 23, 24, 24, 44, 45]. To date, CAA-ri defines a subacute autoimmune encephalopathy, which is presumably caused by increased CSF concentrations of anti-Aβ autoantibodies. This autoinflammatory reaction is both temporally and regionally associated with the formation of amyloid-related imaging abnormalities suggestive of vasogenic edema (ARIA-E), microglial activation and cerebral microbleeds [1, 44, 45] (➔ Fig. 1). The autoinflammatory nature of CAA-ri bears the consequence that CAA-ri responds to immunosuppressive treatment, offering a broad spectrum of therapeutic options [1, 12, 14, 17, 18, 22–24, 27, 47, 54, 55].

In this review, we summarize the existing data on the epidemiological and pathophysiological background of CAA-ri. Additionally, we want to shed light on its variable clinical presentation and the current progress made in the diagnostic workup of CAA-ri. Finally, we critically discuss current results and future directions in immunosuppressive treatment of CAA-ri.

## Main part

### Epidemiology of CAA-ri

To date, large epidemiological studies to estimate the exact prevalence of CAA-ri in the total population are lacking. However, current studies indicate CAA-ri as a disease of the advanced age [1, 2, 20, 52, 63, 64]. This age-dependent prevalence has already found its way into the clinical and radiological diagnostic criteria of CAA-ri [2]. Regarding patient gender, an equivalent distribution is reported [1, 2, 20, 47, 63, 64].

### Etiopathogenesis

The last decade of ongoing research in the field of antibody-mediated amyloid-modifying therapies has triggered increasing interest in the pathomechanisms underlying CAA-ri, due to MR tomographical changes observed in a subset of the treated patients in the bapineuzumab as well as lecanemab trials [58, 59, 65]. These alterations were subsequently termed amyloid-related imaging abnormalities (ARIA) and subdivided into the two groups ARIA-E (vasogenic edemas) and ARIA-H (hemosiderin deposits) [58]. In this context, patients with ARIA-E frequently showed an APOE4 genotype and CSF alterations comparable to CAA-ri [58, 59], hence encouraging the assumption that CAA-ri represented a ‘natural manifestation’ of iatrogenic ARIA-E [22, 58].

The histopathological changes underlying CAA-ri have been known long before the identification of anti-Aβ autoantibodies as a putatively underlying pathophysiologic agent and encompass perivascular multinucleated giant cells alongside with a lymphomonocytic infiltration [18, 20, 48, 62]. Intriguingly, immunohistochemical analysis could show CD68-positive microglial cells adjacent to the affected vessel wall, which have recently been described corresponding to areas of ARIA-E in the (sub)acute phase of CAA-ri [44, 62].

Besides microglial activation and formation of vasogenic edemas, the APOE genotype seems to play a considerable pathophysiological role in CAA-ri development. APOE encodes a 34 kDa glycoprotein expressed in three allelic variants [34]. Based on data from a detrimental [29, 32] resp. favorable [34] APOE4 and APOE2 carrier status in AD patients, such connections have also been investigated in CAA-ri. A current meta-analysis estimates the proportion of APOE4 homozygosity to lie around 34% [63, 64], while little is known about a pathophysiological role of the genes APOE2 and APOE3. Conceivably, the APOE2 genotype might promote a distinct histological subtype with mainly transmural inflammatory infiltrates [18].

### Clinical features

CAA-ri shows a variable clinical presentation, which might occasionally complicate differential diagnosis to other acute neurological, inflammatory or neurodegenerative diseases [55]. Due to impaired vessel integrity, (sub)cortical micro- [1, 2, 13, 17, 18, 23, 24, 44, 45, 51, 54, 63, 64] as well as macrobleeds [1, 2, 8, 20, 47, 63, 64] are favored, which result in focal neurological deficits in about 50% of the patients [1, 47, 63, 64]. Conversely, the vascular Aβ depositions also increase the risk for ischemic strokes [20, 47, 53], though these occur about half as frequent as intracerebral hemorrhages (ICH) [63, 64].

Resulting from the brain-related alterations, about 70% of the patients show progressive cognitive decline [63, 64] with variable clinical presentation [7, 12, 13, 18, 22, 27, 40, 45, 55]. Additionally, more than 50% of the patients develop encephalopathy [23, 24, 44, 45, 51, 54].

Cortical lesions may also increase the risk of epileptic seizures in about one third of CAA-ri patients [40, 47, 63, 64], reaching from focal seizures to status epilepticus [8, 13, 18, 22, 27, 40, 44, 45, 54, 55]. Headache also represents an unspecific leading symptom in CAA-ri [1, 8, 13, 17, 18, 20, 23, 24, 40, 44, 45, 47, 55, 63, 64].

### Establishing the diagnosis of CAA-ri

#### Brain biopsy—the neuropathological point of view

Definite diagnosis of CAA-ri can still only be made by a brain biopsy as the gold standard [18]. Corresponding histopathological analysis shows vascular Aβ depositions comparable to sporadic CAA [2, 7, 18, 20, 21, 26, 27, 35, 48, 51, 62–64]. Histological diagnosis of CAA-ri further requires the identification of a peri- and/ or transmural inflammatory infiltrate surrounding > 1 amyloid-positive vessel, which is made of CD68-positve microglia and T lymphocytes [2, 7, 18, 20, 21, 26, 27, 48, 51, 62].

To date, brain biopsy is only reserved to complex cases, especially for differentiation from brain tumors and for the establishment of definite diagnosis [18, 50, 51]. Hence, a combination of clinical and radiological criteria has been developed to allow diagnosis of CAA-ri in the absence of histopathological confirmation.

#### Clinical and radiological criteria—a pragmatic diagnostic approach

Due to the invasiveness of brain biopsy and to ensure long-term radiological follow-up of diagnosed and treated CAA-ri patients, Kinnecom et al. suggested the implementation of standardized non-invasive criteria to allow diagnosis of CAA-ri [37]. Subsequently, such criteria have been established based on the modified Boston criteria of CAA [15, 30, 38, 39], and have successfully been validated by Auriel et al. [2].

**Fig. 2:**
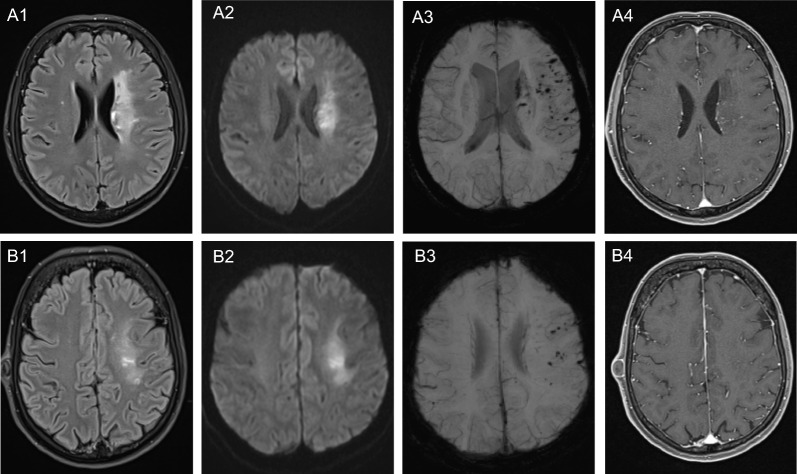
MRI findings in CAA-ri at the level of cella media (**A1**-**A4)** and centrum semiovale (**B1**-**B4)**. FLAIR images (**A1**-**B1)** disclose a left-sided periventricular leukoencephalopathy, with correlating diffusion restriction (**A2**-**B2)**; the DWI signal seems rather indicative of prominent T2-shine through effect than plain stroke. Hemosiderin deposits are depicted (**A3**-**B3)**, extending from the periventricular region to the left frontoparietal cortex. In addition, subtle perivascular enhancement is noted in the periventricular area (**A4**-**B4**)

While definite diagnosis of CAA-ri still requires histopathologic confirmation, these criteria enable the diagnosis of probable and possible CAA-ri using clinical and radiological findings (➔ Table 1). Besides clinical symptoms, the focus lies on cranial MR imaging. According to the STRIVE v1 guidelines [67], the MRI sequences T1 and T2 weighting, FLAIR and DWI as well as susceptibility-weighted imaging (SWI) or alternatively the hem-specificT2* sequence with gradient echo (T2* GRE), should be performed on a regular basis (➔ Fig. 2). Some authors also include a contrast-enhanced T1-weighted (ce T1w) measurement [1, 2, 20, 44, 67]. MR angiography is not yet recommended in CAA-ri, though it might be relevant for the exclusion of differential diagnoses such as RCVS or PACNS.

**Table 1:** Clinicoradiologial diagnostic criteria of CAA-ri established by Auriel et al.

Diagnosis	Criteria
Probable CAA-ri	Patient age ≥ 40 years
	Presence of ≥ 1 of the following clinical features: headache, decrease in consciousness, behavioral change, or focal neurological deficits and seizures; the presentation is not directly attributable to an acute intracerebral hemorrhage
	MRI shows uni- or multifocal WMH lesions extending to the immediate subcortical white matter; the asymmetry is not due to past ICH
	Presence of ≥ 1 of the following corticosubcortical hemorrhagic lesions: cerebral macrobleed, cerebral microbleed, or cortical superficial siderosis
	Exclusion of differential diagnoses (neoplasm, infections etc.)
Possible CAA-ri	Patient age ≥ 40 years
	Presence of ≥ 1 of the following clinical features: headache, decrease in consciousness, behavioral change, or focal neurological deficits and seizures; the presentation is not directly attributable to an acute intracerebral hemorrhage
	MRI shows WMH lesions that extend to the immediate subcortical white matter
	Presence of ≥ 1 of the following corticosubcortical hemorrhagic lesions: cerebral macrobleed, cerebral microbleed, or cortical superficial siderosis
	Exclusion of differential diagnoses (neoplasm, infections etc.)

In the absence of brain biopsy, the clinicoradiological criteria established by Auriel et al. allow the diagnosis of probable (sensitivity 82%, specificity 94%) resp. possible CAA-ri (sensitivity 82%, specificity 69%). For the respective diagnostic category, each of the five criteria has to be fulfilled

The FLAIR sequence predominantly addresses white matter hyperintensities (WMH), which correspond to the vasogenic edemas comparable to ARIA-E [1, 2, 20, 23, 24, 44, 63, 64, 67]. In contrast to CAA, WMH in probable CAA-ri occur asymmetrically (➔ Fig. 2; A1-B1). Regarding their spatial distribution, 89% of WMH show a supratentorial localization [20], though there was no clear preference for a specific brain region [2, 17, 20, 54]. The diagnosis of probable CAA-ri further requires WMH extension to the immediate subcortical white matter. For possible CAA-ri, demonstration of WMH reaching the subcortical white matter independent of their (a) symmetry is sufficient [2]. These changes are frequently associated with a leptomeningeal and/ or parenchymal contrast enhancement [2, 20, 44, 47].

The T2* GRE and SWI sequences allow for identifying ICH and cortical microbleeds (CMB), as well as cortical superficial siderosis (CSS), all of which are characteristics of CAA-ri [2, 18, 63, 64, 67]. CMB represent small SWI/ T2* GRE hypointense lesions, which are caused by parenchymal hemosiderin deposits [67] (➔ Fig. 2; A3-B3). Diagnosis of CAA-ri requires the detection of multiple microbleeds, the numbers of which have been reported ranging from 10 to 480 [1, 20, 63, 64]. CSS defines chronic subarachnoid hemosiderin deposition of variable origin. The T2* GRE sequence shows a linear hyperintensity above the cortex [67].

Altogether, the criteria introduced by Auriel et al. allow diagnosis of probable CAA-ri with a sensitivity of 82% and specificity of 94% compared to a control cohort with sporadic CAA (➔ Table 1). For possible CAA-ri, these values decrease to 82% resp. 69% [2].

#### CSF features

CSF investigation in CAA-ri mainly has its role in the exclusion of differential diagnoses. Several groups have described a lymphocytic pleocytosis in 26–67% of the patients [2, 13, 17, 47, 55, 61, 63, 64], as well as an increased protein concentration (appr. 66–80% of the patients) [2, 13, 18, 47, 55, 63, 64], though both findings are not considered specific for CAA-ri [2, 18, 27, 44, 47, 61]. In most cases, oligoclonal bands are negative in CAA-ri patients [18, 54].

#### Anti-Aβ autoantibodies – game changers in the diagnostic and therapeutic monitoring of CAA-ri?

As stated above, the occurrence of iatrogenic ARIA-E triggered the search for reliable biomarkers to allow risk stratification of AD patients as well as dosage finding of amyloid-modifying therapies [56, 58, 59]. As such, the serum values of anti-Aβ 1–42 antibodies were initially used [19], but yielded conflicting results [7, 12, 20, 45, 49]. Subsequent attempts thus focused on the identification of anti-Aβ autoantibodies in the CSF, which were expected to show a higher specificity. In 2011, DiFrancesco et al. have successfully developed an ultrasensitive ELISA, which demonstrated increased concentrations of anti-Aβ40 and anti-Aβ42 antibodies in the CSF of a single patient with probable CAA-ri compared to healthy controls as well as MS patients. Of note, the autoantibody levels showed marked reduction upon immunosuppressive treatment [22, 31] as well as a specific intrathecal synthesis, hence allowing calculation of their concentration by the Reiber diagram [22]. Additionally, measurements of autoantibody concentrations in the acute versus remission phase of CAA-ri cold reproduce a reduction of the levels upon immunosuppressive treatment [45]. Moreover, Piazza et al. measured the anti-Aβ autoantibody concentration in a single CAA-ri patient over time during corticosteroid therapy. Herein, a progressive reduction of anti-Aβ autoantibody levels upon each steroid pulse could be observed, finally reaching control levels upon remission of CAA-ri [45]. Though low titers of anti-Aβ autoantibodies could also be identified in control cohorts, the concentrations in these groups were significantly lower with an appr. 3- to fourfold concentration difference compared to CAA-ri [45].

Despite the game-changing character of these investigations, the study results raise various questions, which have already partially been addressed within ongoing research in the field. Especially the pronounced heterogeneity of the measured anti-Aβ autoantibody levels in investigated patients raises the question where to define a cutoff value, upon which anti-Aβ autoantibody concentrations are considered pathological [22, 45]. Based on further research, Piazza et al. suggested a threshold of ≥ 32 ng/ml [44]. However, further studies are required to investigate long-term suitability of this value, especially regarding the risk of over- or underdiagnosis and -treatment of CAA-ri patients,and how to deal with borderline results.

Moreover, the question arises whether there is a correlation between anti-Aβ autoantibody titers and CSF concentrations of the neuronal destruction markers. While elevated concentrations were described in an initial analysis by Piazza et al. during the acute phase of CAA-ri [45], these data could not be reproduced in follow-up studies [12, 44].

The recent observations demonstrating increased microglial activation in areas with acute ARIA-E in CAA-ri patients further raise the question, whether there is a direct dose–effect relationship between MR-tomographic alterations and the height of anti-Aβ autoantibody concentrations. Though Piazza et al. could show two patients with severe ARIA-E to have the highest anti-Aβ autoantibody levels during the acute phase of CAA-ri with return to normal levels upon immunosuppressive treatment and regression of MRI hallmarks, the low number of cases does not yet allow statistical analyses regarding significance and/ or correlations between these parameters [44].

Altogether, the anti-Aβ autoantibodies cannot yet be considered as a sole tool for the diagnosis of CAA-ri, but must be interpreted in the overall context of clinical as well as radiological findings. Additionally, measurement of anti-Aβ autoantibody levels has so far not established itself as a comprehensive routine diagnostic test in patients with suspected CAA-ri.

### Differential diagnoses

**Fig. 3:**
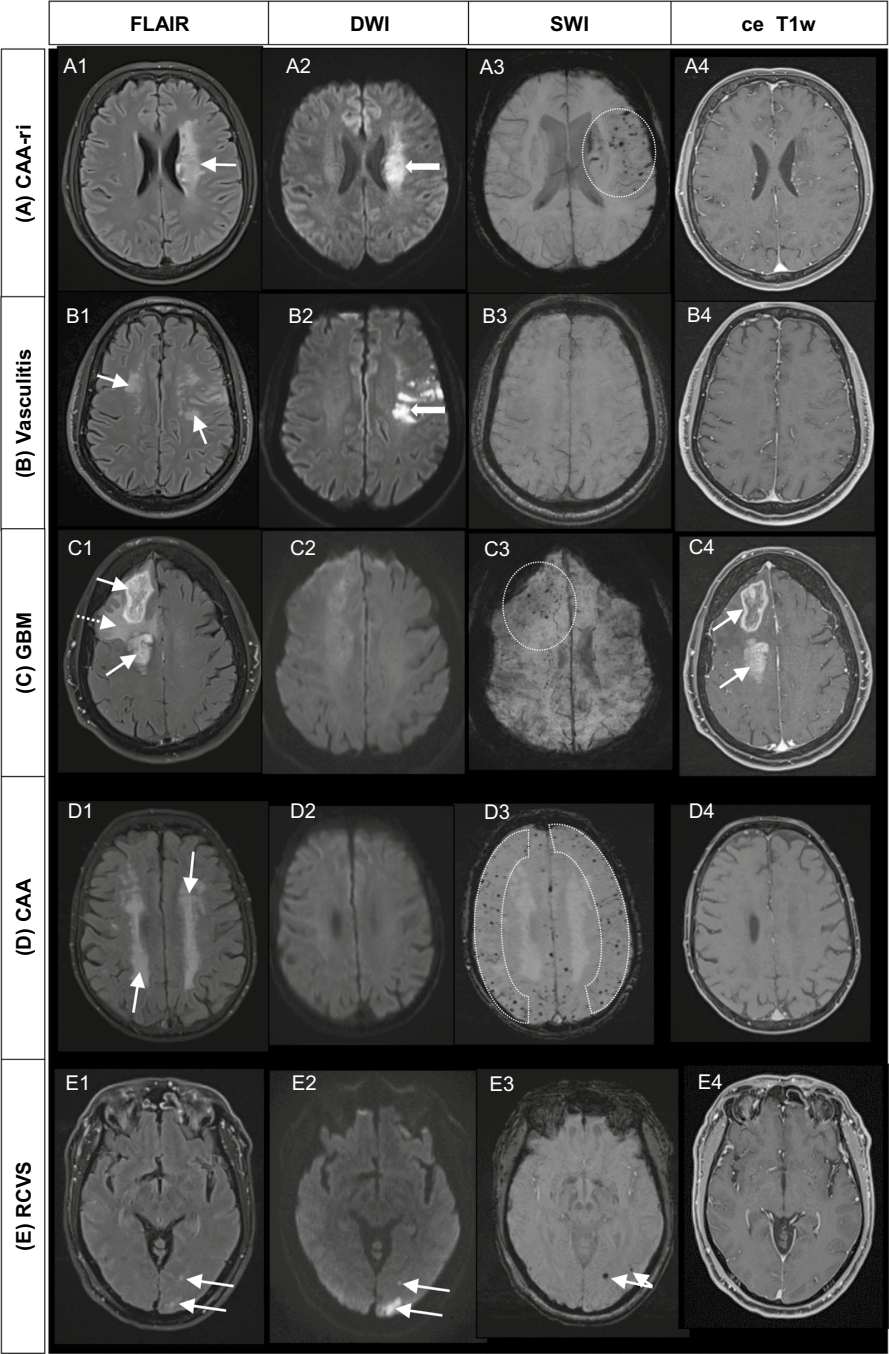
Side-by-side display of imaging findings in CAA-ri as well as in disease mimics. Rows (from top to bottom): MR images of **A** CAA-ri, **B** vasculitis, **C** glioblastoma, **D** CAA and **E** RCVS are shown. Columns (from left to right): Different MRI sequences illustrate the varying pathologies (**A1**-**E1**) FLAIR, (**A2**-**E2**) DWI, (**A3**-**E3**) SWI and (**A4**-**E4**) ce T1w. Whereas regional inflammation (**A1**, arrow) and encephalopathy with microbleeds (**A3**, circle) are a hallmark of CAA-ri, diffuse microbleeds (**D3**, encircled regions) as well as symmetric encephalopathy (**D1**, arrows) are common in CAA. Multifocal leukoencephalopathy (**B1**, arrows) combined with subacute stroke (**B2**, bold arrow) can be expected in vasculitis, thus differing from the rather circumscribed CAA-ri findings. Albeit microbleeds occur in GBM in the context of neovascularization (**C3**, circle), tumorous enhancement and mass lesion character (**C1**, arrows) are untypical findings in CAA-ri. In RCVS, circumscribed FLAIR hyperintensities are depicted in the occipital lobe (**E1**, arrows), with evidence of subacute infarction on DWI images (**E2**, arrows). The SWI sequence discloses punctate hemosiderin spots (**E3**, arrows), whereas no breakdown of the blood–brain barrier is delineated (**E4**)

Due to the similar presentation of CAA-ri to other intracerebral pathologies, careful differential diagnostic checklists also represent part of the clinical as well as diagnostic work-up. Figure 3 and Table 2 summarize the main differential diagnoses of CAA-ri, which can roughly be subdivided into the four categories of vascular, autoinflammatory, infectious and neoplastic diseases.

**Table 2:** Relevant differential diagnoses of CAA-ri

Category	Disease(s)	Clinical + radiological characteristics
Vascular	Sporadic CAA	Diagnosis by modified Boston criteria Symmetric WMH Normal levels of anti-Aβ autoantibodies Neuropathology: absence of peri-/ transmural vascular inflammation
	PRES syndrome	Causes: hypertension, (pre)eclampsia, immunosuppressives, cytotoxic therapies, systemic autoinflammatory diseases FLAIR-hyperintense bilateral symmetric lesions, mainly in occipital lobe $\pm$ posterior parts of the parietal/ temporal lobes and frontal region Mostly subcortical location of lesions
	RCVS	Younger patient cohort (~ 42 years) Thunderclap headache $\pm$ focal neurological signs and epileptic seizures Cerebral angiography: ‘string of beads’ pattern MRI: symmetric reversible brain edema (comparable to PRES), infarctions in ‘watershed regions’, ICH of variable size with cortical predominance, subarachnoid hemorrhage
Autoinflam-matory	PACNS	Younger patient cohort (onset ~ 4th decade) Lumbar puncture: frequent (80–90%) presence of decent lymphocytic pleo-cytosis + elevated protein concentration DSA: possible ‘vessel beading’ MRI: pronounced leptomeningeal en-hancement, multifocal lesions, vessel wall enhancement + thickening in black blood sequences
Neoplastic	Low-grade glioma	T1: solid without enhancement, T2: hyperintense signal DWI: no significant diffusion restriction
Infectious	PML	Hyperintense, multifocal FLAIR lesions, frequently in frontal + parieto-occipital lobes Absence of microbleeds in T2* GRE/ SWI sequence Elevated JC virus PCR results

As stated above, sporadic CAA represents the main vascular differential diagnosis of CAA-ri (➔ Fig. 3; D1-4). Sporadic CAA can be diagnosed using the modified Boston criteria [15, 30, 66] and shows symmetric WMH. Furthermore, investigation of anti-Aβ autoantibody levels [45], or – in complex cases – brain biopsy, provides increasing diagnostic clarity [7, 18, 20, 21, 48, 51, 62].

The posterior reversible encephalopathy syndrome (PRES) and reversible cerebral vasoconstriction syndrome (RCVS) also represent important differential diagnoses of CAA-ri. Both diseases can be summarized as cerebrovascular dysregulation syndromes [57] and may also occur as overlapping phenomena [36, 57]. MR tomographically, PRES is characterized by T2-/ FLAIR-hyperintense, symmetric vasogenic edema with a predominant localization in the posterior as well as occipital lobes, and the frontal region [3, 57, 60]. Corresponding to vasogenic edema, the DWI sequence and ADC map may appear iso- or hyperintense resp. hyperintense [46, 57]. About a sixth of the patients develop vascular complications of the syndrome, whereby ischemic vs. hemorrhagic events occur with a similar frequency [36]. Microbleeds in the T2* GRE/ SWI sequence may occur in PRES [41], albeit found rarely and in a lower amount than in CAA-ri [1, 20, 63, 64]. RCVS occurs in younger patients than CAA-ri (mean patient age: 42 years), frequently presents with ‘thunderclap headache’ [10, 57], and may also show reversible brain edema with FLAIR hyperintensities (➔ Fig. 3, E1), though these do not present with a parieto-occipital predominance as observed in PRES [46]. Intracranial or subarachnoid hemorrhages in RCVS develop more than twice as frequently as ischemic stroke (22% and 20% vs. 15%) [36] (➔ Fig. 3, E2). Subarachnoid hemorrhage in RCVS may occur uni- or bilaterally and can be distinguished from superficial siderosis in CAA-ri by its sulcal FLAIR hyperintensity resp. T2*/ GRE hypointensity near the convexity [16, 25]. Diagnosis of RCVS includes, besides MR imaging, CSF investigation, which may show slight pleocytosis and elevated protein concentrations [25]. Additionally, cerebral angiography demonstrates variable diameter changes along the cerebral vessels (‘sausage on a string’ appearance).

Regarding the autoinflammatory spectrum, primary angiitis of the central nervous system (PACNS) represents the main differential diagnosis of CAA-ri (➔Fig. 3, B1-4). This form of vasculitis mainly occurs in adults around the fourth decade [5]. Nonetheless, CAA-ri and PACNS share common clinical symptoms as well as comparable CSF features, though the latter occur more frequently in PACNS [5] than CAA-ri [2, 27, 44, 47, 61]. Diagnosis of PACNS is made using a combination of CSF features, MR angiography, as well as brain biopsy and digital subtraction angiography (DSA). While DSA may demonstrate ‘vessel beading’ in case of larger vessel affection, brain biopsy may reveal granulomatous or necrotizing vasculitis [5] without Aβ depositions [42] in cases of unremarkable cerebral angiography. MRI sequences recommended for the diagnosis of PACNS correspond to those used in CAA-ri, though PACNS frequently shows pronounced leptomeningeal enhancement, multifocal lesions and vessel wall thickening [5].

The WMH seen in CAA-ri, especially in the context of an underlying mass lesion, may be recognized as low-grade glioma mainly in elderly patients [51] (➔Fig. 3, C1-4). Low-grade gliomas appear solid and without enhancement in T1w, while they show a hyperintense signal in T2 [9]. Furthermore, no significant diffusion restriction and high ADC signals [9], as well as occasionally microbleeds due to tumor-induced neovascularization, can be observed [33].

Additionally, CAA-ri must be differentiated from infectious diseases, especially from progressive multifocal leukoencephalopathy (PML). This disease leads to multifocal patchy FLAIR lesions, which may occur virtually anywhere in the brain [4]. For further differentiation, the SWI/ T2* GRE sequences can be used to identify cerebral microbleeds typical of CAA-ri [2, 18, 67], while elevated JC virus PCR results are considered diagnostic in PML [4].

### Therapy and clinical monitoring of CAA-ri—an overview

Due to the autoinflammatory nature of CAA-ri, the disease shows responsiveness to immunosuppressive therapies [1, 11, 13, 14, 21, 23, 24, 37, 44, 45, 47, 54, 55, 63, 64]. According to data from Antolini and colleagues, sufficient control of autoinflammatory processes might also have a major prognostic impact, as patient outcome seems to be driven solely by the degree of disease activity [1]. At the same time, the chosen immunosuppressive agent does not seem relevant, as clinical improvement occurred almost twice as frequent if CAA-ri patients were treated with any immunosuppressive substance compared to no treatment [47]. Hence, most authors start an initial corticosteroid therapy, though the existing literature shows high heterogeneity regarding the used substances, dosages and forms of application. Common schemes include methylprednisolone [1, 11, 45, 55] or dexamethasone [1, 11, 22, 45]. After the initial pulse therapy, slow oral tapering is recommended to prevent the development of recurrences and to ensure clinical stabilization [1].

Though a current meta-analysis estimates that glucocorticoids are chosen as initial treatment of CAA-ri in up to 75% of the reported cases [63, 64], various authors also use immunosuppressives with a higher potency [63, 64]. In this context, combination therapy of corticosteroids with azathioprine [13, 21, 28, 44, 47, 55] or cyclophosphamide [13, 18, 26, 28, 37, 47, 55] is administered comparatively frequently, though individual therapeutic attempts with mycophenolate mofetil (MMF) [13, 37, 47], methotrexate [21, 47], rituximab [47], or IVIG [14] have also been described.

Despite the wide range of therapeutic options, objective decision criteria for the choice of more aggressive immunosuppressive regimes in the initial as well as follow-up therapy of CAA-ri do not yet exist. While some authors decide on a combination therapy upon first recurrence of CAA-ri [28, 44, 47], it remains matter of debate whether such therapeutic regimes should already be chosen in the initial treatment phase of CAA-ri, following the notion to ‘hit it hard and early’.

Another aspect arising in the context of CAA-ri therapy is the question, when to perform control MR imaging, and how to define clinical and radiological remission resp. relapse of the disease. To date, clinical remission of CAA-ri is defined as complete reconstitution of neurological deficits to the level preceding presentation, or as the sole persistence of neurological deficits due to vascular events caused by CAA-ri [1], while radiologic recovery has been considered fulfilled if a complete resolution resp. decrease or disappearance of WMH and/ or T1 enhancement as well as DWI lesions can be observed [1, 47]. In this context, repetitive assessment of anti-Aβ autoantibodiy titers cannot yet be broadly recommended, as it has only been tested in a single patient to date [45].

These considerations also influence the question, when to conduct control MR imaging. In the largest prospective CAA-ri cohort study to date, the authors propose follow-up MRI after 3, 6, 12, and 24 months following CAA-ri diagnosis. Kaplan–Meier analyses in this study showed that clinical and radiological recovery frequently occur within the first 6 months of treatment (appr. 75–80% of the patients), whereby radiological recovery was slightly delayed to clinical improvement. Additionally, the number of patients at risk showed the strongest reduction within this timespan [1]. Conversely, these data also indicate that approximately a quarter of the patients does not reach remission within this timespan, hence emphasizing the importance of close clinical as well as radiological monitoring within this episode. In a proof-of-concept trial with a small patient cohort, Piazza et al. could demonstrate that clinical and radiological improvement within an average period of 5 months after treatment initiation is also associated with a remarkable reduction of microglial activation within regions of (former) vasogenic edema [44]. However, the ^11^C-PK11195 PET did not show a complete resolution of microglial activation within the 5-month period, though MR tomography yielded complete regression of CAA-ri imaging findings. One could thus speculate that the intervals for radiological follow-up might need to be refined in near future regarding PET findings. Nonetheless, the used ^11^C-PK11195 PET has so far only been tested in this small, specialized study cohort, and has not yet taken its place in routine diagnostics of CAA-ri. Independent of these considerations regarding follow-up imaging, control MRI should always be performed in case of clinical deterioration [44].

## Conclusions

The last years of ongoing research in the field of CAA-ri have opened new perspectives, but have also evoked various questions, which need to be addressed by future studies. Regarding clinical diagnostics of CAA-ri, testing for anti-Aβ autoantibodies in the CSF of affected individuals represents a game changer and should be performed routinely in AD patients preceding administration of amyloid-modifying therapies. Such a procedure might allow dose titration as well as patient stratification regarding the risk of therapy-induced ARIA. However, the technique of anti-Aβ autoantibody determination still needs widespread establishment as well as clarification of various methodological issues.

Additionally, we perceive urgent necessity for further worldwide data collection concerning the diagnostic as well as therapeutic management of newly identified CAA-ri cases to allow the development of therapeutic standards. In near future, rational decision criteria for the choice of more aggressive immunosuppressive regimes in the initial as well as follow-up therapy of CAA-ri need to be defined, which might consider patient characteristics as well as clinical, radiological and CSF anti-Aβ autoantibody findings. Further studies should also answer the question, whether intensification of immunosuppression only in case of clinical and radiological deterioration (corresponding to a ‘treat to target’ approach comparable to MS therapy) or a direct start with highly potent immunosuppressives in sense of a ‘hit it hard and early’ concept represents the right way in the long-term therapy of CAA-ri. These considerations also gain further importance relating to the 20–25% of patients in the study by Antolini et al., which did not reach clinical and radiological recovery after a 6-months follow-up period. It would be interesting to further characterize this subgroup of individuals regarding their clinical, MRI and anti-Aβ autoantibody characteristics to answer the question, whether these patients should already be stratified as ‘high risk’ in initial diagnostics and might be susceptible only to higher immunosuppressive regimes. In this context, further studies might also answer the questions, whether the currently defined MRI control timepoints prove themselves in a larger patient cohort, and how to deal with sole radiological CAA-ri progression without clinical symptoms.

Taken together, CAA-ri is currently considered an ‘orphan disease’. However, this perception should not distract from the fact that current as well as future research on CAA-ri might mark decisive changes in the field of targeted AD therapies. Due to the high prevalence of ARIA in the current amyloid-modifying antibody trials, one might even speculate the ‘true’ prevalence of CAA-ri to be higher than currently supposed.

## Data Availability

Data sharing is not applicable to this article as no datasets were generated or analyzed during the current study.

## Abbreviations

AD: Alzheimer’s disease
ADC: Apparent diffusion coefficient
APOE: Apolipoprotein E
ARIA: Amyloid-related imaging abnormalities
Aβ: Amyloid beta
CAA: Cerebral amyloid angiopathy
CAA-ri: Cerebral amyloid angiopathy-related inflammation
ce T1w: Contrast-enhanced T1-weighted imaging
CMB: Cerebral microbleed
CNS: Central nervous system
CSF: Cerebrospinal fluid
CSS: Cortical superficial siderosis
DSA: Digital subtraction angiography
DWI: Diffusion-weighted imaging
ELISA: Enzyme-linked immunosorbent assay
FLAIR: Fluid-attenuated inversion recovery
GRE: Gradient echo
ICH: Intracerebral hemorrhage
IVIG: Intravenous immunoglobulins
MMF: Mycophenolate mofetil
MR(I): Magnetic resonance (imaging)
MS: Multiple sclerosis
PACNS: Primary angiitis of the central nervous system
PET: Positron emission tomography
PML: Progressive multifocal leukoencephalopathy
PRES: Posterior reversible encephalopathy syndrome
RCVS: Reversible cerebral vasoconstriction syndrome
STRIVE: STandards for ReportIng Vascular changes on nEuroimaging
SWI: Susceptibility-weighted imaging
WMH: White matter hyperintensity
